# *Ehrlichia chaffeensis* Transcriptome in Mammalian and Arthropod Hosts Reveals Differential Gene Expression and Post Transcriptional Regulation

**DOI:** 10.1371/journal.pone.0024136

**Published:** 2011-09-06

**Authors:** Jeeba A. Kuriakose, Simone Miyashiro, Tian Luo, Bing Zhu, Jere W. McBride

**Affiliations:** 1 Department of Pathology, Center for Biodefense and Emerging Infectious Diseases, University of Texas Medical Branch, Galveston, Texas, United States of America; 2 Department of Microbiology and Immunology, University of Texas Medical Branch, Galveston, Texas, United States of America; 3 Center for Biodefense and Emerging Infectious Diseases, University of Texas Medical Branch, Galveston, Texas, United States of America; 4 Sealy Center for Vaccine Development, University of Texas Medical Branch, Galveston, Texas, United States of America; 5 Institute for Human Infections and Immunity, University of Texas Medical Branch, Galveston, Texas, United States of America; Tulane University, United States of America

## Abstract

**Background:**

Human monocytotropic ehrlichiosis is an emerging life-threatening zoonosis caused by obligately intracellular bacterium, *Ehrlichia chaffeensis. E. chaffeensis* is transmitted by the lone star tick, *Amblyomma americanum*, and replicates in mononuclear phagocytes in mammalian hosts. Differences in the *E. chaffeensis* transcriptome in mammalian and arthropod hosts are unknown. Thus, we determined host-specific *E. chaffeensis* gene expression in human monocyte (THP-1) and in *Amblyomma* and *Ixodes* tick cell lines (AAE2 and ISE6) using a whole genome microarray.

**Methodology/Principal Findings:**

The majority (∼80%) of *E. chaffeensis* genes were expressed during infection in human and tick cells. There were few differences observed in *E. chaffeensis* gene expression between the vector *Amblyomma* and non-vector *Ixodes* tick cells, but extensive host-specific and differential gene expression profiles were detected between human and tick cells, including higher transcriptional activity in tick cells and identification of gene subsets that were differentially expressed in the two hosts. Differentially and host-specifically expressed ehrlichial genes encoded major immunoreactive tandem repeat proteins (TRP), the outer membrane protein (OMP-1) family, and hypothetical proteins that were 30–80 amino acids in length. Consistent with previous observations, high expression of p28 and OMP-1B genes was detected in human and tick cells, respectively. Notably, *E. chaffeensis* genes encoding TRP32 and TRP47 were highly upregulated in the human monocytes and expressed as proteins; however, although TRP transcripts were expressed in tick cells, the proteins were not detected in whole cell lysates demonstrating that TRP expression was post transcriptionally regulated.

**Conclusions/Significance:**

*Ehrlichia* gene expression is highly active in tick cells, and differential gene expression among a wide variety of host-pathogen associated genes occurs. Furthermore, we demonstrate that genes associated with host-pathogen interactions are differentially expressed and regulated by post transcriptional mechanisms.

## Introduction

Human monocytotropic ehrlichiosis (HME) is a life-threatening emerging tick-borne zoonosis caused by obligately intracellular bacterium, *Ehrlichia chaffeensis* [1]. HME is a systemic disease characterized by clinical presentation that includes fever, headache, myalgia, anorexia, chills and laboratory abnormalities including leucopenia, thrombocytopenia, anemia and elevation of serum hepatic aminotransferases [1]. The severity of the disease varies from asymptomatic seroconversion to a fatal multisystem failure [2]. *E. chaffeensis* is transmitted by the lone star tick, *Amblyomma americanum,* and maintained in nature by persistent infection of mammalian hosts [1]. In the mammalian host, *E. chaffeensis* replicates primarily within mononuclear phagocytes forming membrane-bound cytoplasmic microcolonies called morulae that are resistant to innate immune destruction [3].

Bacterial pathogens survive by expressing genes necessary for transmission, invasion and persistence, and evasion of innate and adaptive defenses [4]. Among these include surface proteins of *Borrelia burgdorferi* and *Yersinia pestis*, secreted effectors of *Shigella flexneri* and transcriptional regulator of *Bordetella pertussis* [5]–[7]. Moreover, host-specific gene expression by *Anaplasma phagocytophilum* has been reported in human and tick cells [8], and the *E. chaffeensis* p28 outer membrane protein encoded by the OMP-1 multigene locus is differentially expressed in human and tick cells [9]–[11]. Furthermore, it is recognized that *E. chaffeensis* propagated in tick cells has a distinct antigen expression profile from that of mammalian phagocyte grown ehrlichiae [12].

*E. chaffeensis* has a relatively small genome (1.18 Mbp) [13], but has evolved within mammalian and arthropod hosts and developed mechanisms to subvert host immune defenses. There are numerous *Ehrlichia* genes that are associated with host-pathogen interactions [14], including tandem repeat (TRPs) and ankyrin repeat proteins (Anks), actin polymerization proteins, poly (G–C) tracts, Type IV secretion (T4S) system and a multigene family encoding the outer membrane proteins (OMP-1) that exhibit porin activity [15], [16]. TRPs (TRP120, TRP47 and TRP32) and Anks (Ank200) elicit strong antibody responses in the mammalian host and have major continuous species-specific antibody epitopes in acidic domains that include the serine-rich tandem repeats [17]–[19]. The TRPs are secreted, and TRP47 and TRP120 are differentially expressed on the surface of dense-cored (infectious) ehrlichiae [18]–[20].

Molecular interactions between TRP47 and the mammalian host identified numerous host cell targets with distinct cellular functions associated with signaling, transcriptional regulation, vesicle trafficking and cellular proliferation and differentiation [21]. TRP120 has been shown to play an important role in binding and internalization [22], and its expression is regulated by the second messenger cyclic di-GMP and protease HtrA [23]. It is also associated with novel molecular protein-protein, protein-DNA interactions suggesting that it is involved in modulating host cell processes and gene transcription [24], [25]. *E. chaffeensis* Ank200 was recently detected in the mammalian host cell nuclei and interacts with an adenine-rich motif in promoter and *Alu* elements [26].

The macrophage transcriptome during *E. chaffeensis* infection has been previously determined [27]; however, investigation of *E. chaffeensis* gene expression in distinct hosts has been limited to genes encoding the OMP-1 multigene family. In this study, we analyzed the *E. chaffeensis* transcriptome in human monocytes (THP-1), tick cells from the known arthropod vector (*A. americanum*; AAE2 cells) and the vector of *A. phagocytophilum* (*Ixodes scapularis*; ISE6 cells) and determined that well characterized ehrlichial proteins involved in host-pathogen interactions were differentially expressed.

## Results

### *E. chaffeensis* genes expressed in THP-1, AAE2 and ISE6 cells

The transcriptome of *E. chaffeensis* in THP-1 consisted of 79% of all genes (n = 1031). Similar expression levels were observed in AAE2 (76%) and ISE6 (81%).

### Differentially expressed *E. chaffeensis* genes

**Figure 1:**
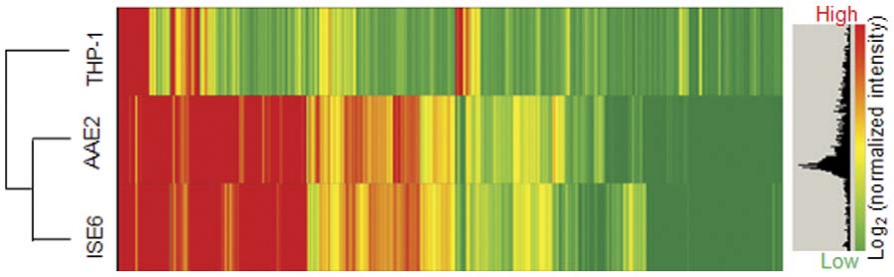
Microarray data of gene expression profiles from *E. chaffeensis-*infected human and tick cell lines. Heat map with gene expression in THP-1, AAE2 and ISE6 cells, coloring: red, up-regulated; yellow, normal; green, down-regulated.

**Figure 2:**
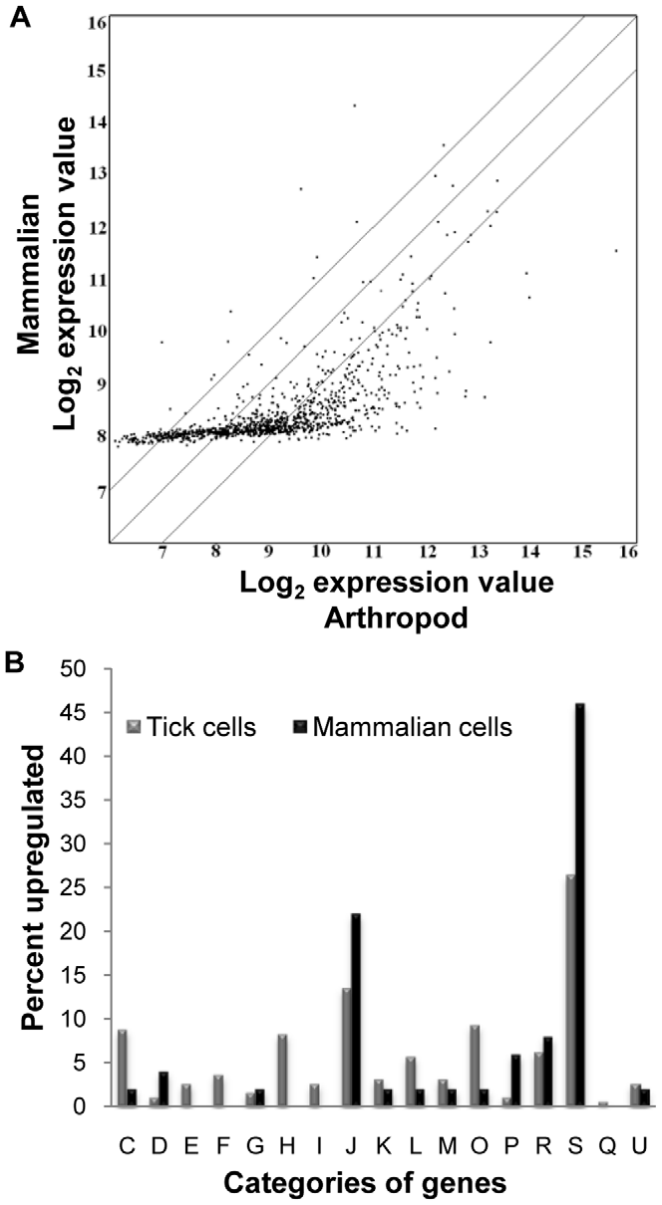
Genes upregulated by *E. chaffeensis* in human and tick cells. (A) Scatter plot of expression in mammalian (THP-1) vs. arthropod (AAE2 and ISE6) cells, center line represents equivalence and outer lines indicate two fold difference. Images generated using ArrayStar®. (B) Distribution of significantly upregulated *E. chaffeensis* genes in mammalian cells (black bars) and tick cells (grey bars) classified to Clusters of Orthologous Groups (COGs). C: Energy production and conversion, D: Cell cycle control and mitosis, E: Amino acid metabolism and transport, F: Nucleotide metabolism and transport, G: Carbohydrate metabolism and transport, H: Coenzyme metabolism, I: Lipid metabolism, J: Translation, K: Transcription, L: Replication and repair, M: Cell wall/membrane/envelope biogenesis, O: Post-translational modification, protein turnover, chaperone functions, P: Inorganic ion transport and metabolism, R: General functional prediction only, S: Function unknown, Q: Secondary structure, U: Intracellular trafficking and secretion.

*E. chaffeensis* genes were differentially expressed in THP-1 compared to AAE2 and ISE6 cells. Minor differences in *E. chaffeensis* gene expression between the tick cell lines were observed (Fig. 1). There were 405 *E. chaffeensis* genes (39%) differentially expressed (greater than 2 fold change; *p*<0.005) between THP-1 and ISE6 cells, 371 genes (36%, *p*<0.005) differentially expressed between THP-1 and AAE2, and 351 were similarly expressed in the tick cell lines (Fig. 2A).

### *E. chaffeensis* genes upregulated in the human monocytes

There were 50 *E. chaffeensis* genes upregulated (>2 fold; *p*<0.05) in the THP-1 cells compared to both AAE2 and ISE6 cells (Table 1), and 19 additional genes upregulated in THP-1 compared to AAE2 cells. In contrast, only five additional genes were upregulated in THP-1 compared to ISE6 cells. When classifying the genes according to the Clusters of Orthologous Groups (COGs) [28], the *E. chaffeensis* genes upregulated in the THP-1 cells were grouped into the metabolic and cellular process (C, G, P, Q, D); transcription, translation and DNA repair (J, K, L); cell envelope biogenesis and outer membrane (M); posttranslational modifications (O); general function predicted or unknown (R,S); trafficking and secretion (U) (Fig. 2B, black bars). The majority of these genes (54%) were classified as hypothetical with unknown functions.

**Table 1:** *E. chaffeensis* genes upregulated in THP-1 cells compared to AAE2 and ISE6 cells.

Gene Function	Gene ID	Fold Change THP-1 vs(AAE2, ISE6)>2fold; *p*<0.05
**Function Unknown or Predicted**		
1. Conserved hypothetical protein	ECH_0147	5.3, 4.8
2. Conserved hypothetical protein	ECH_0619	5.6, 5.3
3. Conserved hypothetical protein	ECH_0790	2.6, 2.6
4. Conserved hypothetical protein	ECH_1059	2.3, 2.4
5. Conserved hypothetical protein	ECH_1122	7.4, 6.7
6. Hypothetical protein	ECH_0034	2.6, 2.4
7. Hypothetical protein	ECH_0078	3.2, 3.1
8. Hypothetical protein	ECH_0253	3.2, 2.2
9. Hypothetical protein	ECH_0254	3.7, 2.2
10. Hypothetical protein	ECH_0265	3.3, 2.0
11. Hypothetical protein	ECH_0682	2.1, 2.5
12. Hypothetical protein	ECH_0685	2.6, 2.7
13. Hypothetical protein	ECH_0833	2.9, 3.1
14. Hypothetical protein	ECH_0834	3.1, 2.6
15. Hypothetical protein	ECH_0887	3.9, 2.0
16. Hypothetical protein	ECH_0909	2.8, 2.1
17. Hypothetical protein	ECH_0921	2.3, 2.2
18. Hypothetical protein	ECH_0965	2.2, 2.2
19. Hypothetical protein	ECH_1049	3.7, 4.1
20. Hypothetical protein	ECH_1056	4.8, 2.6
21. Hypothetical protein	ECH_1102	4.5, 2.5
22. Conserved hypothetical protein (TRP47)	ECH_0166	11.4, 14.1
23. Variable length PCR target protein (TRP32)	ECH_0170	9.4, 7.7
24. HAD-superfamily hydrolase, subfamily IA, variant 1	ECH_0332	2.2, 2.2
25. Putative flavin reductase	ECH_0442	2.5, 2.8
26. Putative NADH dehydrogenase I, J subunit, truncation	ECH_0550	3.1, 2.7
27. Rhodanese domain protein	ECH_0896	2.2, 2.0
**Cell envelope biogenesis/Outer membrane**		
28. Major outer membrane protein P28	ECH_1143	2.2, 2.2
**Trafficking/Secretion**		
29. Preprotein translocase, SecG subunit	ECH_0172	4.2, 4.3
**Posttranslational modification/Protein turnover/Chaperones**		
30. Trigger factor	ECH_0902	2.3, 2.0
**Transcription/Translation/DNA replication/RNA**		
31. Cold shock protein, CSD family	ECH_0298	2.9, 2.7
32. DNA-binding protein HU	ECH_0804	2.1, 2.6
33. Putative ribosomal protein S18	ECH_0309	3.2, 2.9
34. Ribosomal protein L13	ECH_1019	3.5, 3.4
35. Ribosomal protein L15	ECH_0427	2.3, 2.0
36. Ribosomal protein L20	ECH_0197	2.5, 2.7
37. Ribosomal protein L34	ECH_0440	2.8, 2.5
38. Ribosomal protein L35	ECH_0198	5.7, 5.1
39. Ribosomal protein S13	ECH_0430	2.2, 2.3
40. Ribosomal protein S15	ECH_0727	4.6, 4.0
41. Ribosomal protein S19	ECH_0413	2.2, 2.5
42. Ribosomal protein S6	ECH_0308	2.6, 2.4
43. Ribosomal protein S8	ECH_0423	2.2, 2.1
**Metabolism/Cellular Processes**		
44. ATP synthase F1, delta subunit	ECH_0131	2.4, 2.5
45. Cell division protein FtsA	ECH_1090	2.2, 2.1
46. Monovalent cation/proton antiporter, MrpF/PhaF subunit family	ECH_0466	2.7, 2.3
47. Na(+)/H(+) antiporter subunit C	ECH_0469	2.7, 2.2
48. Ribose 5-phosphate isomerase B	ECH_0638	3.4, 2.9
49. Superoxide dismutase, Fe	ECH_0493	2.6, 2.6
50. YGGT family protein	ECH_0891	3.1, 3.3

### *E. chaffeensis* genes upregulated in the tick cells

There were 193 *E. chaffeensis* genes upregulated (>2 fold; *p*<0.05) in tick cells compared to human cells (Table S1). The largest proportion (32%) belonged to the COG with general function predicted or unknown (R, S), 30% were involved in metabolism and cellular process, and 7% of the genes were associated with translation (J) (Fig. 2B, grey bars). The remaining *E. chaffeensis* genes (31%) were distributed in the other COGs.

### Hyper-expressed genes in the human monocytes

There were ten *E. chaffeensis* genes expressed in the THP-1 cells with expression levels 10–15 times higher (hyper-expressed) than other genes identified as highly expressed. These genes included TRP47 (the highest expressed gene), TRP32, ribosomal proteins, malonyl CoA-acyl carrier protein transacylase, and hypothetical proteins (ECH_0166, ECH_1059, ECH_0570, ECH_0253).

### Expression of genes associated with host-pathogen interactions

For the two *E. chaffeensis* proteins recently shown to bind mammalian host cell DNA, transcripts for TRP120 and Ank200 genes were detected in human and tick cells. The *E. chaffeensis* genome encodes for a polymorphic multigene family composed of 22 paralogues that are clustered in a 29 kb gene locus that is downstream of the transcriptional regulator gene *tr1* [29]. Transcriptional regulator *tr1* (ECH_1118) of *E. chaffeensis* was expressed in human and tick and cells. With respect to the OMP-1 (p28) family genes, transcripts were not detected for OMP-1H (p28–11) and OMP-1W (p28–7) in human cells and OMP-1P (p28–3), OMP-1U (p28–5) and OMP-1H (p28–11) were not detected in tick cells, but OMP-1B (p28–14) and OMP-1N (p28–1) were hyper-expressed. Notably, OMP-1B (p28–14) was up-regulated in tick cells, but OMP-1N (p28–1) was also highly expressed in human cells. OMP-1F (p28–18) and OMP-1D (p28–16) transcripts were also upregulated in tick cells compared to human cells, and P28 (p28–19) was among the most highly expressed OMP-1 genes in human cells.

*E. chaffeensis* genes associated with protein trafficking and secretion were expressed in tick and human cells; however, several of these genes were upregulated in the tick cells compared to human cells, including SecF, TatC, TatA and members of the type IV secretion systems. Additionally, in the tick cells, several of the genes associated with posttranslational modification, and protein turnover were upregulated including several ATP-dependent proteases and chaperones.

### Expression of hypothetical genes

A large percentage (42%) of annotated *E. chaffeensis* genes encode hypothetical proteins with unknown functions [13]. In this study, we determined that most of these genes were differentially expressed in human and tick cell lines. There were 27 hypothetical genes (COG; R, S) that were highly expressed in the THP-1 cells, including TRP32 and TRP47 (Table 1). However, 11 of these 27 genes were not expressed by *E. chaffeensis* in the AAE2 and ISE6 cells (Table 2). Most of these genes (9/11) encoded peptides (30–70 amino acids) that do not have orthologs. Host-specific expression of these genes suggested that they are required exclusively for adaptation and survival within the mammalian host.

**Table 2:** Hypothetical genes expressed only in human (THP-1) cells.

Gene Function	SEQ_ID	Length (Amino Acids)	Predicted Cellular Location
Hypothetical protein	ECH_0790	44	Cytoplasmic
Hypothetical protein	ECH_0034	31	Cytoplasmic
*Hypothetical protein	ECH_0078	56	Cytoplasmic
Hypothetical protein	ECH_0353	73	Cytoplasmic
Hypothetical protein	ECH_0833	50	Cytoplasmic
Hypothetical protein	ECH_0834	49	Cytoplasmic
Hypothetical protein	ECH_0921	52	Cytoplasmic
Hypothetical protein	ECH_0965	203	Inner Membrane
*Hypothetical protein	ECH_1056	47	Cytoplasmic
Hypothetical protein	ECH_1113	48	Cytoplasmic
*Hypothetical protein	ECH_0887	39	Cytoplasmic

*Expression values 3–5 times greater than average expression values.

There were a larger number of *E. chaffeensis* genes that were differentially expressed in the tick cells, including 63 genes categorized as hypothetical (COG; R, S). Some of these genes (n = 18) were expressed only in the tick cells (Table 3). Of these genes, ECH_0114, ECH_0249, ECH_0258, ECH_0889, ECH_1030, ECH_1048 were highly expressed (expression values 3–5 times greater than the average expression value). Of the 18 genes differentially expressed in the tick cells, seven did not have orthologs and six were peptides (30–80 aa). ECH_0114 was predicted to be a secreted protein, and ECH_0526 and ECH_1038 were predicted as outer membrane proteins (CELLO subcellular localization predictor) [30]. In a previous study, ECH_0526 protein expression was detected in both AAE2 and ISE6 tick cell lines [31].

**Table 3:** Hypothetical genes expressed only in tick (AAE2 and ISE6) cells.

Gene Function	SEQ_ID	Length (Amino Acids)	Predicted Cellular Location
Conserved hypothetical protein	ECH_0516	120	Cytoplasmic
Conserved hypothetical protein	ECH_0767	621	Cytoplasmic
Conserved hypothetical protein	ECH_0988	208	Cytoplasmic
Conserved hypothetical protein	ECH_1154	135	Cytoplasmic
Conserved domain protein	ECH_0526	495	Outer Membrane
Hypothetical protein	ECH_0059	49	Cytoplasmic
Hypothetical protein	ECH_0099	42	Cytoplasmic
*Hypothetical protein	ECH_0114	122	Extracellular
*Hypothetical protein	ECH_0249	46	Cytoplasmic
*Hypothetical protein	ECH_0258	35	Cytoplasmic
Hypothetical protein	ECH_0635	357	Cytoplasmic
Hypothetical protein	ECH_0765	79	Cytoplasmic
Hypothetical protein	ECH_0868	31	Cytoplasmic
*Hypothetical protein	ECH_0889	38	Cytoplasmic
*Hypothetical protein	ECH_1030	62	Cytoplasmic
Hypothetical protein	ECH_1038	1963	Outer Membrane
*Hypothetical protein	ECH_1048	64	Cytoplasmic
Hypothetical protein	ECH_1077	68	Cytoplasmic

*Expression values 3–5 times greater than average expression values.

### Validation of microarray data

**Figure 3:**
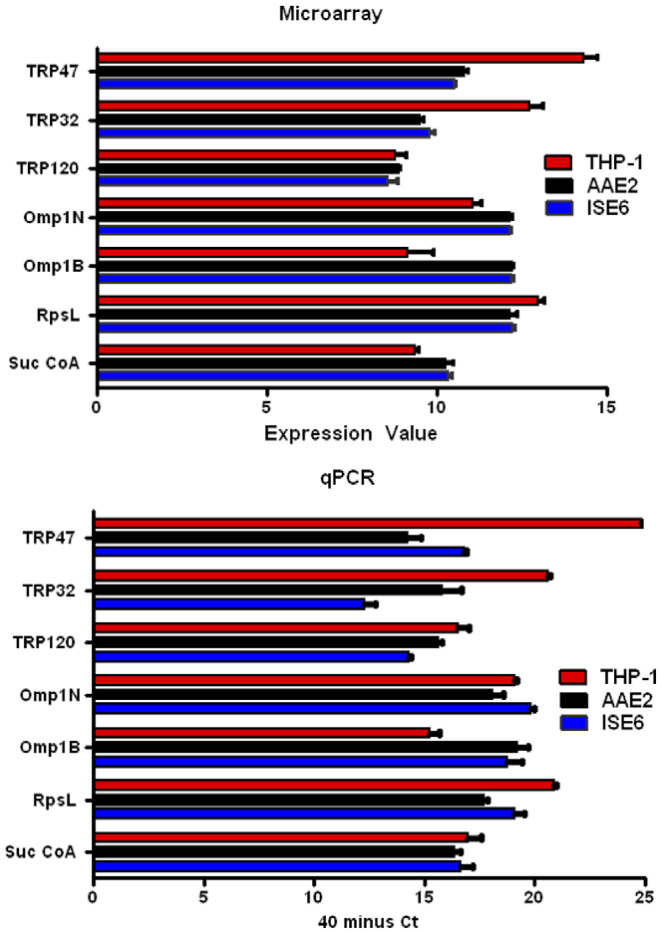
Comparison of microarray (gene expression value) and qPCR (40 minus threshold cycle) analysis of selected *E. chaffeensis* transcript levels.

Real-time quantitative RT-PCR was used to verify the microarray results of a subset of ehrlichial genes. Eight *E. chaffeensis* genes assayed for relative transcript abundance by qRT-PCR, included; Suc CoA (ECH_0979), RpsL (ECH_0963), OMP-1B (ECH_1136), OMP-1N (ECH_1121), TRP120 (ECH_0039), TRP32 (ECH_0170), TRP47 (ECH_0166). The relative transcripts levels for the selected genes within and between cell lines confirmed expression levels determined by microarray (Fig. 3).

### Expression of tandem repeat proteins

**Figure 4:**
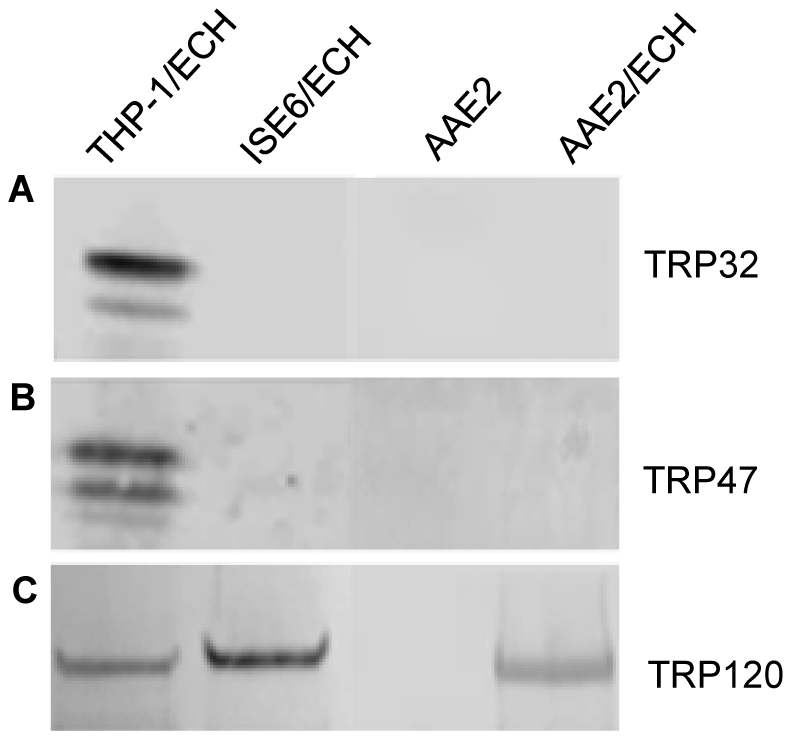
Expression of *E. chaffeensis* TRPs in human (THP-1) and tick (ISE6 and AAE2) cell lysates. Western immunoblots were probed with anti-TRP32 (A), anti-TRP47 (B) and anti-TRP120 (C) antibodies. TRP120 was detected in *E. chaffeensis*-infected human and tick cell lysates, and TRP32 and TRP47 were detected only in *E. chaffeensis*-infected THP-1 cells.

Expression of three tandem repeat proteins; TRP32, TRP47 and TRP120 were analyzed in AAE2, ISE6 and THP-1 cells. TRP120 transcript was detected in all three cell lines, and the protein was also expressed in human monocytes and tick cells (Fig. 4). Interestingly, transcripts for hyper-expressed genes TRP32 and TRP47 were detected in tick cells by microarray and qRT-PCR; however, these proteins were not detected by western immunoblot in *E. chaffeensis*-infected AAE2 and ISE6 cell lysates (Fig. 4).

## Discussion

Defining the dynamic changes in pathogen gene and protein expression that occurs in infected hosts is essential to understanding pathobiology and having a rational basis for vaccine development. This investigation was conducted because of our lack of knowledge regarding the relative *E. chaffeensis* gene expression in mammalian and arthropod hosts, which is a major impediment to understanding which genes are essential for ehrlichial adaptation. In this study, we demonstrated that the expression of many *E. chaffeensis* genes was influenced by the host environment. In addition, we examined *E. chaffeensis* gene expression in tick cell lines from the established vector species and another common tick that is not a vector. Significant differences in *E. chaffeensis* gene expression were not observed between the two tick cell lines, and similar expression patterns were observed in *E. chaffeensis* genes involved in metabolic and cellular processes between human and tick cells. Differentially expressed genes identified were primarily hypothetical genes and genes associated with translation and posttranslational modification. Furthermore, we have also found evidence of post-transcriptional regulation of select ehrlichial genes involved in host-pathogen interactions in mammalian and arthropod hosts.

In mammalian cells, the *Ehrlichia* developmental cycle occurs within 72 hrs and is characterized by entry of the dense-cored form, replication of the reticulate cells and transformation to infectious dense-cored ehrlichiae [32]. In this study, enriched bacterial RNA was extracted from *E. chaffeensis* infected THP-1, AAE2 and ISE6 cells when 90% of the cells were infected. Although the infection was not synchronized to evaluate a specific phase of the developmental cycle, cells were harvested when 90% of the cells had *E. chaffeensis* morulae within their cytoplasm; therefore the data presented in this study likely includes genes expressed during all phases of development, but may be more representative of ehrlichial transcription during the later developmental stages that is dominated by dense-cored ehrlichiae [32].

The *I. scapularis* cell line, ISE6, has been routinely used in previous investigations involving arthropod-borne pathogens including *Ehrlichia*, *Rickettsia*, *Anaplasma* and *Borrelia* [33]; however, *I. scapularis* is not a natural vector of *E. chaffeensis*. Recently, Munderloh *et. al*, developed the *A. americanum*, AAE2 cell line from tick embryos (Munderloh, U. and Davidson, W.R., unpublished data), and *E. chaffeensis* protein expression in AAE2 tick cells has been investigated [31], but a comprehensive analysis of *E. chaffeensis* gene expression has not been determined in the AAE2 cell line. Therefore, we investigated gene expression in both cell lines to determine if significant differences existed. Notably, we did not identify any significant differences in *E. chaffeensis* gene expression; hence, it appears that either cell line could be used for vector-pathogen studies for *E. chaffeensis*. However, the AAE2 cells grew more rapidly and appeared to support more robust growth of ehrlichiae, which are also important considerations.

Transcripts were detected for ∼80% of the *E. chaffeensis* genes in human monocytes and tick cells. This level of transcript detection is slightly higher (∼10%) than that previously reported for *A. phagocytophilum* in human (HL-60) and tick (ISE6) cells [8]. This difference may be related to the fact that we utilized pathogen-enriched RNA rather than total RNA. Most *E. chaffeensis* genes (81–95%) involved in metabolic and cellular process, transcription, translation, DNA repair, cell envelope biogenesis, outer membrane proteins, posttranslational modifications, general function predicted or unknown, trafficking and secretion were expressed in all three cell lines. There were 77 genes for which transcripts were not detected in any of the three cell lines, and the function of the majority of these genes is unknown. It is possible that these genes are required during stages of infection not depicted in this study such as transmission of the pathogen from one host to the other, reactivation of the pathogen after a blood meal in the tick, or in the presence of tick saliva.

Although similar numbers of genes were expressed by *E. chaffeensis* in the human and tick cells, the most striking discovery is that 38% of the *E. chaffeensis* genes were differentially expressed. When compared to human cells, *E. chaffeensis* was transcriptionally more active in the tick cells, and there were a larger number of genes with high expression levels in the tick cells. The functions of these genes were associated with protein modification, energy production and conversion and nutrient transport. Similar genes were upregulated in *Rickettsia conorii* and *R. rickettsii* under conditions of nutrient limitations and lower temperatures, when the metabolism of the host cells slows [34], [35]. In contrast, the majority of the genes had a moderate expression level in human cells. Additionally, there were several genes involved in metabolism, cellular process, and translation that were upregulated in tick cells. The upregulation of these genes in the arthropod host suggests that *Ehrlichia* has higher metabolic activity in the tick. The number of genes differentially expressed by *E. chaffeensis* between the two host cells was similar to that observed for *A. phagocytophilum* [8]. However, only minimal differences were observed in gene expression when *R. rickettsii* grown in ISE6 was compared to *R. rickettsii* grown in Vero cells [35]. Although all three organisms have evolved to adapt within both arthropod and mammalian cells, there appears to be significant differences between *Rickettsia* compared to *Ehrlichia* and *Anaplasma* suggesting that they have different adaptation mechanisms and pathobiology.

In contrast to the total expression level (∼80%) of genes from the other functional groups, the 437 *E. chaffeensis* genes with unknown function were expressed at a lower level (∼67%) in each cell line and the majority were differentially expressed. We identified 11 genes highly expressed in the human cells that were not expressed in the tick cells and identified 18 genes exclusively expressed in the tick cells, and the majority of these hypothetical genes encoded peptides, 30-80 amino acids in length. Additionally, these peptides are *E. chaffeensis*-specific and do not have orthologs in other ehrlichial species. Nearly half of the genes (n = 243) annotated as hypothetical proteins in the *E. chaffeensis* genome contain fewer than 100 amino acids, and recently peptides were detected for 66% of these proteins during infection in THP-1 cells [36]. Host-induced expression of these *E. chaffeensis* peptides suggests that they are required exclusively for adaptation and survival within the mammalian host. Further studies are needed to characterize these genes and their role in host-specific adaption and survival.

In human cells, there were ten ehrlichial genes that were hyper-expressed, defined as genes with expression values 10–15 times greater than other highly expressed genes. Among these genes were two major immunoreactive tandem repeat proteins, TRP47 and TRP32. TRP47, the most highly expressed *E. chaffeensis* gene in human cells, contains seven 19-mer tandem repeats that dominate the C-terminal region and several N-terminal tyrosine phosphorylation sites [21], [37]. A recent study to examine molecular interactions between TRP47 and the host identified several interactions with specific host cell proteins that have distinct cellular functions associated with signalling, transcriptional regulation, vesicle trafficking, and cellular proliferation and differentiation [21]. The hyper-expression of TRP47 in human cells, the absence of TRP47 in tick cells, and our recent findings regarding molecular host-pathogen interactions, suggests that TRP47 is a multifunctional effector that is required for ehrlichial intracellular survival within the mammalian host. Unlike TRP47 which is differentially expressed by the dense-cored form of *E. chaffeensis*, TRP32 is extracellularly associated with the morular fibrillar matrix and the morula membrane, indicating that this protein is secreted. TRP32 does not have homology with other known proteins [18]; however, we have recently demonstrated that TRP32 interacts with proteins with functions similar to those that interact with TRP47 [38]. In the tick cells, although transcripts were detected for the TRP47 and TRP32, the proteins were not detected suggesting that they are regulated posttranslationally.

*E. chaffeensis* TRP120 is a well characterized protein that is differentially expressed on the surface of the dense-cored *E. chaffeensis*. Similar levels of TRP120 transcripts were detected in human and tick cells, and the protein was detected in both cell lysates. Our findings regarding TRP120 expression were in contrast to a previous study that examined macrophage- and tick cell-derived proteins of *E. chaffeensis*, in which TRP120 was not detected in macrophages, but was detected in tick cell lysates [31]. However, numerous other studies have reported TRP120 expression in ehrlichiae cultivated in human cells [20], [22], [23]. TRP120 has known functional properties including binding and internalization, and its surface expression is regulated by second messenger cyclic di-GMP and interacts with host cell proteins associated with biological processes similar to TRP47 [22]–[24]. Furthermore, we recently, demonstrated that TRP120 binds host cell DNA and targets genes associated with biological processes known to be altered during *E. chaffeensis* infection [25]. Although TRP120 has important functions in the mammalian host related to pathobiology, the role of TRP120 in the arthropod host is unknown. The expression of TRP120 in the tick cells suggests that it may have similar functions in the arthropod host.

The OMP-1/P28 multigene family of *E. chaffeensis* have been well studied and host cell-specific expression of these genes has been previously reported [10], [11]. The function of these immunoreactive outer membrane proteins has been associated with immune evasion; however, Rikihisa *et. al.* recently demonstrated porin activity for OMP-1F and P28 [16], [39], [40], suggesting an important functional role in nutrient acquisition. Consistent with previous studies, we determined that Omp-1B and p28 were expressed in human and tick cells. The upregulation of p28 (p28–19) in human cells and the high expression of OMP-1B (p28–14) in tick cells were also consistent with previous *in vitro* studies [11], [12], and expression of OMP-1B (p28–14) transcript has been detected in all three developmental stages of the tick vector, *A. americanum* [11]. However, our finding that OMP-1N (p28–1) was upregulated in tick cells has not been previously reported. Transcripts were not detected for OMP-1H (p28–11) and OMP-1W (p28–7) in the human cells. The absence of OMP-1W (p28–7) expression was also consistent with the fact that it could not be detected in dogs experimentally infected with *E. chaffeensis* [11]. However, OMP-1H (p28–11) was detected in experimentally infected dogs and DH82 cells (canine cell line) [11], [41], but not in the human and tick cells suggesting that there are other host factors that contribute to expression of OMP-1H. Similarly, although transcripts for OMP-1 family members have been detected in several studies, OMP-1B is the only OMP-1 paralogue detected by proteomics in *E. chaffeensis* cultured in ISE6 cells [31]. The role of host-specific OMP expression is not clear, but our findings suggest that vaccines targeting ehrlichial OMPs expressed in the tick should include OMP-1N. Host cell specific expression of these genes could be related to adaptation to different host environments and for nutrient acquisition.

It is generally recognized that regulation of bacterial gene expression is controlled by transcriptional and posttranscriptional mechanisms [42]–[44]. Several recent studies have investigated the mechanisms involved in the mRNA and protein stability and translational regulation in prokaryotes, and their dependence on environmental conditions and growth phase, especially with virulence factors [45], [46]. Bacterial protein expression is not only dependent on levels of mRNAs but also on other RNA species. Regulatory RNAs such as small RNAs (sRNA) controlling virulence and pathogenesis have been demonstrated in other Gram-negative bacteria including *Escherichia coli*, *Pseudomonas aeruginosa*, *Salmonella typhimurium*, and *Chlamydia trachomatis* [47], [48]. In this investigation, transcripts were detected for TRP47 and TRP32 in the tick cells, yet the proteins were not detected suggesting that their expression is, in part, controlled by posttranslational mechanisms in response to host cell environments, potentially by regulatory RNAs. Similarly, OMP-1B (P28–14) transcripts have been routinely detected in mammalian cells, yet the protein has not been detected in numerous proteomic studies [9], [11], [31], [49]. Therefore, there is evidence that posttranslational mechanisms are involved in TRP expression and could also be involved in regulating OMP expression. Further investigation of posttranslational regulation mechanisms in *Ehrlichia* survival in mammalian and arthropod hosts is needed to understand how ehrlichial protein expression is regulated and its role in host adaptation.

Understanding the molecular survival strategies within the distinct hosts and the mechanisms involved in host adaptation will lead to novel prophylactic and therapeutic targets to prevent transmission and infection. We determined that some TRPs, OMPs, and hypothetical proteins are differentially expressed, and thus, appear to be important for adaptation to each host. Additionally, the hyper-expression of the TRP32 and TRP47 genes in the human cells and absence of the expression of these proteins in the tick cells demonstrate their significance in the mammalian host. The subset of *E. chaffeensis* hypothetical genes identified exclusively in each of the host cells in this study should be examined and their functions determined.

## Methods

### Cell culture and cultivation of *E. chaffeensis*

**Figure 5:**
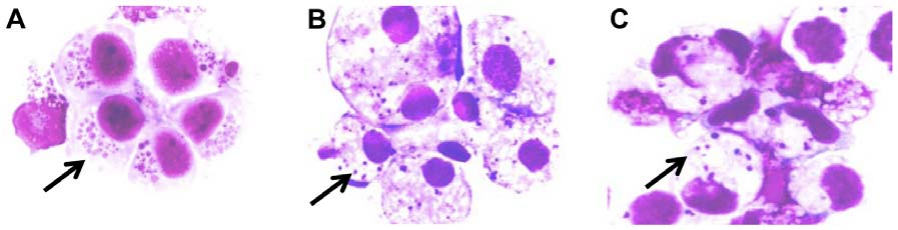
*E. chaffeensis* morulae in (A) THP-1, (B) AAE2, and (C) ISE6 cells stained with Giemsa stain (100x; identified by arrows).

*E. chaffeensis* (Arkansas strain) was cultivated in THP-1 cells, a human monocytic leukemia cell line (ATCC# TIB-202, Manassas, VA) and tick cells (AAE2 and ISE6). THP-1 cells were cultured in Dulbecco's modified Eagle's medium (Invitrogen, Carlsbad, CA) supplemented with 10% fetal bovine serum (HyClone, Logan, UT), 1% HEPES buffer (Sigma, St. Louis, MO), 1% sodium pyruvate (Sigma) at 37°C in a humidified 5% CO_2_ atmosphere. Uninfected AAE2 and ISE6 cells and *E. chaffeensis*-infected tick cells were obtained from Dr. Ulrike Munderloh (University of Minnesota) and were maintained in L15B300 medium supplemented with 10% fetal bovine serum (Harlan, Indianapolis, IN), 10% tryptose phosphate broth (BD, Sparks, MD) and 1% bovine lipoprotein cholesterol concentrate (MP Biomedicals, Irvine, CA) at 34°C as previously described [50], [51]. Uninfected cells were propogated in T-150 flasks, and *E. chaffeensis* infection was maintained in the cells by subculturing with infected cells (10%) to uninfected cells. The level of ehrlichial infection was assessed by modified Giemsa stained (HEMA 3, Fisher Scientific) cytocentrifuged cells (Fig. 5).

### RNA extraction

Total RNA was purified from uninfected and *E. chaffeensis*-infected (90% infected) THP-1, AAE2 and ISE6 cells (10^7^cells per sample) using Tri reagent (Ambion, Austin, TX). Genomic DNA was eliminated by treatment with Turbo DNA-free (Ambion) according to the manufacturer's protocol. Polyadenylated host mRNA was removed using oligo (dT) columns (Oligotex, Qiagen, Valencia, CA) as previously described [52], and bacterial RNA was enriched using Terminator (Epicenter Biotechnologies, Madison, WI) that selectively digests RNAs with 5′-monophosphates that are present only on ribosomal RNA. RNA concentration was determined by NanoDrop (Thermo Scientific, Wilmington, DE) and quality confirmed by Agilent 2100 Bioanalyzer (Agilent Technologies, Santa Clara, CA) at the UTMB Genomics Core Facility. The RNA quality requirements were established as: A260/A280 ≥1.8, A260/A230 ≥1.8 and concentrations ≥ 1 µg/ul.

### Microarray design

The genome sequence of *E. chaffeensis* (GenBank accession no. CP000236) [13] was submitted to Roche NimbleGen Systems (Madison, WI) for custom 4-plex microarray design. The arrays were manufactured using maskless, digital micromirror technology. Five replicates of the genome were included per chip, with an average of 12 different 60-base oligonucleotides (60-mer probes) representing each open reading frame in the *E. chaffeensis* genome. Unique probes were designed for 1031 of the 1158 ORFs (1.18 Mbp genome). Three biological replicates were included for *E. chaffeensis* cultivated in each cell line (THP-1, AAE2 and ISE6). Additionally, for each cell line, RNA was extracted from uninfected cells (negative controls) and was processed similarly, and these samples were used to establish background subtraction thresholds.

### Hybridization

Enriched *E. chaffeensis* RNA (10 µg) was used for cDNA synthesis using random hexamer primers and the SuperScript Double-Stranded cDNA Synthesis Kit (Invitrogen) according to the NimbleGen Arrays User's Guide (Gene Expression Analysis v3.2). Labeling and hybridization was done at the MD Anderson Cancer Center, Genomics Facility (Houston, TX). Briefly, double-stranded cDNA was random-prime labeled with Cy3-nonamers and hybridized to the microarray for 16 hrs at 42°C. The arrays were washed, dried and scanned using a GenePix 400B microarray scanner (Molecular Devices, Sunnyvale, CA).

### Microarray data analysis

Data were extracted from the scanned array images using NimbleScan software (Roche NimbleGen). Quantile normalization was performed across replicates within the 4-plex arrays, and RMA (Robust Multichip Average) analysis was performed to generate gene expression values [53]. The genes expressed were determined by subtracting expression values obtained from uninfected cells from those of infected cells from the same cell line. Analysis and visualization of the expression data were performed using ArrayStar4 software (DNASTAR Inc., Madison, WI), using mean log_2_ expression values for the three biological replicates for each cell line. F-test (ANOVA) was used to compare the mean gene expression values for replicates (within same cell line) and groups of replicates (between cell lines) for a given gene. The microarray data generated in this study have been deposited in NCBI's Gene expression Omnibus [54]. The data are accessible through GEO series accession number GSE29109 (http://www.ncbi.nlm.nih.gov/geo/query/acc.cgi?acc=GSE29109).

### Real time quantitative PCR

Real time PCR of selected *E. chaffeensis* genes was performed with gene specific primers designed using Lasergene 8 (DNASTAR) (Table 4). RNA (1 µg) was used as template for cDNA synthesis using iScript cDNA synthesis kit (Bio-Rad Laboratories, Hercules, CA) according to the manufacturer's instructions. qPCR was performed using iQ SYBR Green supermix (Bio-Rad), gene-specific primers and thermal cycling protocol that consisted of an initial denaturation step of 95°C for 2 min, and 40 cycles of 95° for 10 s, 55°C for 30 s, and 65°C for 30 s using a Mastercycler EP Realplex^2^ S (Eppendorf). DNA from infected cells was used as positive control. Samples lacking cDNA and cDNA from uninfected cells were used as negative control. qPCR data were converted by subtracting the Ct value from the number of cycles (40 cycles) to obtain values.

**Table 4:** Primers for qRT-PCR.

Gene	SEQ_ID	Primers (5′-3′)	Amplicon Size (bp)
Suc CoA	ECH_0979	F –ATTAGGCGAAGTTGATGGTR-TCTTTTGAGGTCTGATGAGTAAT	186
RpsL	ECH_0963	F-CGCGTGTAAAAATTGCTGGTTR-CGCGCCTTCTTCCTATTTTG	186
Omp1B	ECH_1136	F-AACGACAGCAGAGAAGGCR-AACAGGACAGATCCAGCC	183
Omp1N	ECH_1121	F-TCATGTTTAGGATTTGGAGTAR-GTGGCTTGCTGTTTGGT	220
TRP120	ECH_0039	F-ATGGATATTGATAATAGTAACATAAGTACR-TGTGTCATCTTCTTGCTCTTG	162
TRP32	ECH_0170	F- TGATTCACATGAGCCTTCTCR-GGACCAGGTAACTCAACAAA	303
TRP47	ECH_0166	F-CGTGGTGTACAAGCTGAAAR-CTCTTCCGTGTGCATCAT	202

### Western immunoblotting

Whole cell lysates (1 µg) from uninfected and *E. chaffeensis*-infected (90% infected) THP-1, AAE2 and ISE6 cells were separated by sodium dodecyl sulfate-polyacrylamide gel electrophoresis (SDS-PAGE), transferred to nitrocellulose membranes, and western immunoblotting was performed as previously described [55] using rabbit anti-TRP32, anti-TRP47, or anti-TRP120 antibodies [18]–[20]. Bound primary antibodies were detected with alkaline phosphatase-conjugated anti-rabbit IgG (H+L) secondary antibody (Kirkegaard & Perry Laboratories, Gaithersburg, MD) and visualized after incubation with BCIP/NBT (5-bromo-4-chloro-3-indolylphosphate-nitroblue tetrazolium) substrate.

## Supporting Information

Table S1 ***E. chaffeensis*** **genes upregulated in AAE2 and ISE6 compared to THP-1 cells.**
(DOC)
