# Supplementary material for: Ehrlichia chaffeensis Transcriptome in Mammalian and Arthropod Hosts Reveals Differential Gene Expression and Post Transcriptional Regulation
Source: PLoS One. 2011 Sep 6;6(9):e24136. doi: 10.1371/journal.pone.0024136 (PMC3167834; doi:10.1371/journal.pone.0024136)
Supplement: Table S1 — E. chaffeensis genes upregulated in AAE2 and ISE6 compared to THP-1 cells. (DOC) [file pone.0024136.s001.doc]

Table S1. *E. chaffeensis* genes upregulated in AAE2 and ISE6 compared to THP-1 cells.

| **Gene Function** | **Gene ID** | **Fold Change AAE2, ISE6 vs**  **THP-1>2fold; *p*<0.05** |
| --- | --- | --- |
| **Function Unknown or Predicted** |  |  |
| 1. Conserved domain protein | ECH_0526 | 2.1, 2.3 |
| 1. Conserved domain protein | ECH_0894 | 3.2, 2.0 |
| 1. Conserved hypothetical protein | ECH_0397 | 5.3, 4.2 |
| 1. Conserved hypothetical protein | ECH_0477 | 2.7, 3.3 |
| 1. Conserved hypothetical protein | ECH_0540 | 2.0, 2.8 |
| 1. Conserved hypothetical protein | ECH_0663 | 3.0, 2.6 |
| 1. Conserved hypothetical protein | ECH_0706 | 2.8, 3.2 |
| 1. Conserved hypothetical protein | ECH_0725 | 3.6, 3.9 |
| 1. Conserved hypothetical protein | ECH_0767 | 2.3, 2.5 |
| 1. Conserved hypothetical protein | ECH_0836 | 2.1, 2.5 |
| 1. Conserved hypothetical protein | ECH_0854 | 3.0, 2.1 |
| 1. Conserved hypothetical protein | ECH_0988 | 2.6, 2.6 |
| 1. Conserved hypothetical protein | ECH_1148 | 2.2, 2.1 |
| 1. Hypothetical protein | ECH_0059 | 2.1, 2.3 |
| 1. Hypothetical protein | ECH_0114 | 5.7, 5.8 |
| 1. Hypothetical protein | ECH_0117 | 2.8, 4.7 |
| 1. Hypothetical protein | ECH_0150 | 4.3, 4.2 |
| 1. Hypothetical protein | ECH_0158 | 4.5, 3.1 |
| 1. Hypothetical protein | ECH_0159 | 3.7, 3.0 |
| 1. Hypothetical protein | ECH_0191 | 4.2, 4.0 |
| 1. Hypothetical protein | ECH_0205 | 5.3, 7.4 |
| 1. Hypothetical protein | ECH_0242 | 5.2, 8.9 |
| 1. Hypothetical protein | ECH_0244 | 4.4, 4.8 |
| 1. Hypothetical protein | ECH_0258 | 5.8, 5.8 |
| 1. Hypothetical protein | ECH_0270 | 3.8, 3.9 |
| 1. Hypothetical protein | ECH_0271 | 4.1, 3.4 |
| 1. Hypothetical protein | ECH_0272 | 2.7, 2.3 |
| 1. Hypothetical protein | ECH_0284 | 3.3, 2.6 |
| 1. Hypothetical protein | ECH_0285 | 5.4, 5.5 |
| 1. Hypothetical protein | ECH_0288 | 3.2, 4.1 |
| 1. Hypothetical protein | ECH_0329 | 2.8, 3.2 |
| 1. Hypothetical protein | ECH_0349 | 2.5, 2.0 |
| 1. Hypothetical protein | ECH_0388 | 3.0, 3.9 |
| 1. Hypothetical protein | ECH_0531 | 4.8, 3.7 |
| 1. Hypothetical protein | ECH_0535 | 2.6, 2.1 |
| 1. Hypothetical protein | ECH_0722 | 2.5, 2.0 |
| 1. Hypothetical protein | ECH_0723 | 3.6, 2.6 |
| 1. Hypothetical protein | ECH_0765 | 2.1, 2.1 |
| 1. Hypothetical protein | ECH_0825 | 2.3, 2.0 |
| 1. Hypothetical protein | ECH_0829 | 2.0, 2.0 |
| 1. Hypothetical protein | ECH_0878 | 5.1, 4.7 |
| 1. Hypothetical protein | ECH_0916 | 2.7, 3.1 |
| 1. Hypothetical protein | ECH_0925 | 2.0, 2.1 |
| 1. Hypothetical protein | ECH_0927 | 2.0, 2.2 |
| 1. Hypothetical protein | ECH_1048 | 6.0, 5.6 |
| 1. Hypothetical protein | ECH_1092 | 3.7, 4.5 |
| 1. Hypothetical protein | ECH_1104 | 2.8, 3.1 |
| 1. Peptidase, M16 family | ECH_0235 | 4.7, 2.5 |
| 1. Phage portal protein, HK97 family | ECH_0033 | 2.6, 3.4 |
| 1. Phospholipase/carboxylesterase family protein | ECH_0935 | 2.2, 2.4 |
| 1. P-loop hydrolase family protein | ECH_0008 | 2.6, 2.7 |
| 1. Putative flavoprotein | ECH_0061 | 2.0, 2.2 |
| 1. Putative membrane protein, TIGR00023 | ECH_0027 | 2.6, 2.8 |
| 1. Putative osmotically inducible protein | ECH_0335 | 3.6, 2.4 |
| 1. Putative oxidoreductase | ECH_0213 | 4.1, 6.1 |
| 1. Putative phosphatidate cytidylyltransferase | ECH_0269 | 2.9, 2.3 |
| 1. Putative surface protein | ECH_0188 | 2.3, 2.3 |
| 1. Smr domain protein | ECH_0797 | 2.4, 4.1 |
| 1. Tim44-like domain protein | ECH_0232 | 8.4, 13.1 |
| 1. Acid phosphatase SurE | ECH_0791 | 3.8, 2.2 |
| 1. Competence/damage-inducible protein CinA C-terminal domain | ECH_0005 | 3.7, 5.1 |
| 1. Exopolysaccharide synthesis protein | ECH_0769 | 2.6, 2.8 |
| 1. Hemolysin | ECH_0031 | 4.2, 4.0 |
| **Cell envelope biogenesis/Outer membrane** |  |  |
| 1. Major outer membrane protein OMP-1B | ECH_1136 | 8.3, 8.2 |
| 1. Major outer membrane protein OMP-1F | ECH_1142 | 3.1, 2.9 |
| 1. Major outer membrane protein Omp-1N | ECH_1121 | 2.1, 2.1 |
| 1. Outer membrane protein, OmpH family | ECH_1072 | 3.0, 3.1 |
| 1. Putative membrane-associated zinc metalloprotease | ECH_1070 | 3.8, 2.1 |
| 1. D-alanyl-D-alanine carboxypeptidase family protein | ECH_1067 | 2.8, 3.0 |
| **Trafficking/Secretion** |  |  |
| 1. Protein-export membrane protein SecF | ECH_0095 | 3.0, 2.8 |
| 1. Sec-independent protein translocase protein TatC | ECH_0560 | 2.7, 4.9 |
| 1. Twin-arginine translocation protein, TatA | ECH_0844 | 3.4, 2.5 |
| 1. Type IV secretion system protein, VirB6 family | ECH_0497 | 2.2, 2.2 |
| 1. Type IV secretion system protein,VirB6 family | ECH_0498 | 2.5, 3.8 |
| **Posttranslational modification/Protein turnover/Chaperones** | | |
| 1. ATP-dependent Clp protease, ATP-binding subunit ClpB | ECH_0367 | 2.0, 2.3 |
| 1. ATP-dependent Clp protease, ATP-binding subunit ClpX | ECH_0900 | 2.2, 2.5 |
| 1. ATP-dependent metalloprotease FtsH | ECH_1098 | 3.0, 2.6 |
| 1. ATP-dependent protease HslV | ECH_0996 | 3.4, 2.6 |
| 1. ATP-dependent protease La | ECH_0899 | 2.4, 2.3 |
| 1. Chaperone protein HtpG | ECH_0853 | 2.3, 2.1 |
| 1. Chaperonin, 10 kDa | ECH_0364 | 3.4, 5.5 |
| 1. Co-chaperone GrpE | ECH_0168 | 2.5, 4.0 |
| 1. Glutaredoxin 3 | ECH_1062 | 4.3, 4.1 |
| 1. Glycoprotease family protein | ECH_0730 | 5.2, 5.3 |
| 1. Heat shock protein HslVU, ATPase subunit HslU | ECH_0997 | 3.9, 3.8 |
| 1. Heme exporter protein CcmC | ECH_0321 | 4.8, 2.8 |
| 1. HflC protein | ECH_1051 | 2.2, 2.1 |
| 1. NifU domain protein | ECH_0202 | 4.9, 6.8 |
| 1. Rotamase family protein | ECH_0731 | 2.9, 2.3 |
| 1. Signal peptide peptidase SppA | ECH_0401 | 2.1, 2.4 |
| 1. Thioredoxin-disulfide reductase | ECH_0735 | 4.5, 3.9 |
| 1. Zinc finger-like domain protein | ECH_0057 | 2.7, 2.3 |
| **Transcription/Translation/DNA replication/RNA** |  |  |
| 1. Ankyrin repeat protein | ECH_0877 | 2.1, 2.1 |
| 1. Ankyrin repeat protein | ECH_0653 | 5.5, 2.9 |
| 1. Aspartyl-tRNA synthetase | ECH_0334 | 2.3, 2.3 |
| 1. Conserved hypothetical protein | ECH_0803 | 2.8, 4.4 |
| 1. Cysteinyl-tRNA synthetase | ECH_0768 | 3.1, 4.0 |
| 1. DNA gyrase, A subunit | ECH_0858 | 2.5, 2.3 |
| 1. DNA-directed RNA polymerase, beta subunit | ECH_0952 | 2.6, 2.2 |
| 1. DNA-directed RNA polymerase, beta' subunit | ECH_0951 | 2.3, 2.5 |
| 1. Glutamyl-tRNA synthetase | ECH_0605 | 2.3, 2.0 |
| 1. GTP-binding protein YchF | ECH_0154 | 6.3, 4.1 |
| 1. Holliday junction DNA helicase RuvB | ECH_0319 | 2.6, 2.5 |
| 1. Integration host factor, alpha subunit | ECH_0162 | 8.2, 10.0 |
| 1. Leucyl-tRNA synthetase | ECH_0794 | 2.0, 2.2 |
| 1. Lysyl-tRNA synthetase | ECH_0626 | 2.9, 2.0 |
| 1. Methionyl-tRNA formyltransferase | ECH_0897 | 3.3, 2.2 |
| 1. Methionyl-tRNA synthetase | ECH_1000 | 2.0, 2.0 |
| 1. N utilization substance protein A | ECH_0562 | 3.6, 2.5 |
| 1. Peptide deformylase | ECH_0073 | 2.6, 2.3 |
| 1. Phenylalanyl-tRNA synthetase, beta subunit | ECH_0434 | 2.4, 2.7 |
| 1. PolyA polymerase/tRNA nucleotidyltransferase family protein | ECH_1116 | 2.8, 3.0 |
| 1. Polyribonucleotide nucleotidyltransferase | ECH_0726 | 4.6, 2.9 |
| 1. Primosomal protein N' | ECH_0483 | 2.8, 2.6 |
| 1. Putative methyltransferase | ECH_0211 | 2.5, 4.0 |
| 1. Replicative DNA helicase | ECH_0451 | 2.0, 2.3 |
| 1. Ribonuclease III | ECH_1054 | 2.5, 2.7 |
| 1. Ribosomal protein L1 | ECH_0955 | 2.1, 2.8 |
| 1. Ribosomal protein L14 | ECH_0419 | 2.8, 3.0 |
| 1. Ribosomal protein L17 | ECH_0433 | 3.2, 2.4 |
| 1. Ribosomal protein L21 | ECH_0545 | 3.7, 4.0 |
| 1. Ribosomal protein L24 | ECH_0420 | 4.6, 5.9 |
| 1. Ribosomal protein L3 | ECH_0409 | 2.8, 4.0 |
| 1. Ribosomal protein L4 | ECH_0410 | 2.4, 3.2 |
| 1. Ribosomal protein L7/L12 | ECH_0953 | 3.2, 5.1 |
| 1. Ribosomal protein S5 | ECH_0426 | 2.3, 4.6 |
| 1. Ribosomal protein S9 | ECH_1018 | 8.3, 11.8 |
| 1. Ribosomal RNA large subunit methyltransferase J | ECH_0533 | 3.5, 2.1 |
| 1. RNA methyltransferase, TrmH family, group 3 | ECH_0404 | 2.1, 2.5 |
| 1. RNA polymerase sigma factor RpoD | ECH_0760 | 3.1, 2.6 |
| 1. Single-strand binding protein | ECH_0815 | 4.0, 2.3 |
| 1. Single-stranded-DNA-specific exonuclease RecJ | ECH_1115 | 2.6, 2.3 |
| 1. Site-specific recombinase, phage integrase family | ECH_0341 | 2.6, 2.6 |
| 1. Sua5/YciO/YrdC/YwlC family protein | ECH_0802 | 2.3, 2.3 |
| 1. tRNA (5-methylaminomethyl-2-thiouridylate)-methyltransferase | ECH_0872 | 2.1, 2.2 |
| **Metabolism/Cellular Processes** |  |  |
| 1. 2-amino-4-hydroxy-6- hydroxymethyldihydropteridine-pyrophosphokinase | ECH_0350 | 3.3, 2.0 |
| 1. 3-demethylubiquinone-9 3-methyltransferase | ECH_0637 | 3.3, 2.0 |
| 1. 3-oxoacyl-(acyl-carrier-protein) synthase II | ECH_0882 | 5.1, 3.5 |
| 1. 3-oxoacyl-(acyl-carrier-protein) synthase III | ECH_0448 | 4.4, 2.4 |
| 1. Adenosylmethionine-8-amino-7-oxononanoate aminotransferase | ECH_0666 | 3.7, 2.9 |
| 1. Adenylosuccinate synthetase | ECH_0461 | 4.5, 2.6 |
| 1. Amidophosphoribosyltransferase | ECH_0139 | 3.8, 3.4 |
| 1. Argininosuccinate lyase | ECH_0937 | 2.3, 2.4 |
| 1. ATP synthase F0, B chain | ECH_1089 | 3.1, 2.5 |
| 1. Biotin--acetyl-CoA-carboxylase ligase | ECH_0848 | 2.4, 2.3 |
| 1. Chromosome partitioning ATPase, ParA family | ECH_1156 | 2.1, 2.1 |
| 1. Coproporphyrinogen III oxidase, aerobic, truncation | ECH_0592 | 5.2, 3.2 |
| 1. Cytochrome C, membrane-bound | ECH_0327 | 4.6, 3.6 |
| 1. Deoxyuridine 5'triphosphate nucleotidohydrolase | ECH_0501 | 2.3, 2.1 |
| 1. Diaminopimelate epimerase | ECH_0050 | 4.4, 5.3 |
| 1. Dihydrolipoamide dehydrogenase | ECH_0509 | 3.2, 2.6 |
| 1. Dioxygenase family protein | ECH_0368 | 6.1, 5.7 |
| 1. Divalent ion tolerance protein CutA1 | ECH_0756 | 2.3, 2.3 |
| 1. DNA / pantothenate metabolism flavoprotein family protein | ECH_0374 | 2.6, 2.4 |
| 1. Fatty acid/phospholipid synthesis protein PlsX | ECH_0447 | 4.7, 3.4 |
| 1. Ferredoxin A | ECH_0038 | 6.5, 8.8 |
| 1. Ferrochelatase | ECH_0395 | 7.2, 7.2 |
| 1. FeS cluster assembly scaffold IscU | ECH_0630 | 4.9, 4.4 |
| 1. FolD bifunctional protein | ECH_0324 | 2.5, 2.2 |
| 1. Folylpolyglutamate synthase | ECH_0702 | 3.2, 3.2 |
| 1. Fumarate hydratase, class II | ECH_0376 | 3.0, 2.6 |
| 1. Glucose inhibited division protein A | ECH_0359 | 3.3, 3.2 |
| 1. GMP synthase | ECH_0123 | 5.2, 3.0 |
| 1. HIT family protein | ECH_0826 | 4.1, 4.5 |
| 1. Inorganic pyrophosphatase | ECH_1014 | 9.6. 6.1 |
| 1. Major facilitator family transporter | ECH_0816 | 2.2, 2.3 |
| 1. NADH dehydrogenase I, B subunit | ECH_0787 | 5.7, 5.0 |
| 1. NADH dehydrogenase I, H subunit | ECH_0617 | 2.6, 2.8 |
| 1. NADH dehydrogenase I, I subunit | ECH_0691 | 6.0, 3.7 |
| 1. NADH dehydrogenase I, K subunit | ECH_0553 | 2.2, 2.0 |
| 1. NADH dehydrogenase I, L subunit | ECH_0554 | 2.0, 2.0 |
| 1. NADH:ubiquinone oxidoreductase family protein | ECH_0184 | 2.3, 3.6 |
| 1. NADH-ubiquinone/plastoquinone oxidoreductase family protein | ECH_0328 | 3.6, 3.8 |
| 1. Ornithine carbamoyltransferase | ECH_0077 | 6.1, 8.0 |
| 1. Phosphomethylpyrimidine kinase | ECH_0914 | 2.0, 2.0 |
| 1. Phosphoribosylamine--glycine ligase | ECH_1006 | 3.2, 2.1 |
| 1. Porphobilinogen deaminase | ECH_0701 | 2.2, 2.2 |
| 1. Propionyl-CoA carboxylase, alpha subunit | ECH_0487 | 2.6, 2.1 |
| 1. Propionyl-CoA carboxylase, beta subunit | ECH_0599 | 3.5, 2.1 |
| 1. Putative phosphoribosylformylglycinamidine synthase I | ECH_0362 | 2.4, 2.3 |
| 1. Putative pyruvate dehydrogenase complex, E1 component, beta subunit | ECH_0149 | 3.7, 2.3 |
| 1. Pyridoxamine 5'-phosphate oxidase | ECH_0931 | 3.3, 3.0 |
| 1. Riboflavin biosynthesis protein RibD | ECH_0169 | 2.5, 2.7 |
| 1. Riboflavin synthase, alpha subunit | ECH_0239 | 4.3, 2.6 |
| 1. Ribulose-phosphate 3-epimerase | ECH_0082 | 6.5, 6.9 |
| 1. Serine hydroxymethyltransferase | ECH_0311 | 4.2, 3.1 |
| 1. Succinate dehydrogenase, flavoprotein subunit | ECH_0315 | 3.2, 2.6 |
| 1. Succinyl-CoA synthetase, alpha subunit | ECH_0980 | 3.6, 2.6 |
| 1. Succinyl-diaminopimelate desuccinylase | ECH_0144 | 2.1, 2.0 |
| 1. Thiamin biosynthesis protein ThiC | ECH_0798 | 3.5, 5.0 |
| 1. Thiamin biosynthesis ThiG | ECH_0206 | 6.6, 3.3 |
| 1. Transketolase | ECH_0465 | 4.1, 4.3 |
| 1. Uroporphyrinogen decarboxylase | ECH_0030 | 3.4, 5.8 |
